# Ray Resection for Recurrent Invasive Squamous Cell Carcinoma: A Case Report

**DOI:** 10.51894/001c.14612

**Published:** 2020-10-30

**Authors:** Josiah Valk, Brittany Valk, Matthew Caid, Alexander Colen, Richard Singer

**Affiliations:** 1 Orthopaedic Surgery Beaumont Health System; 2 Dermatology Beaumont Health System; 3 Orthopaedic Surgery, Adult Reconstruction Northwell Health System; 4 Orthopaedic Surgery, Hand Surgery University at Buffalo School of Medicine and Biomedical Sciences; 5 Orthopaedic Surgery, Hand Surgery Beaumont Health System

**Keywords:** hand surgery, finger amputation, ray resection, squamous cell carcinoma, orthopaedic oncology

## Abstract

Squamous cell carcinoma is the most common tumor of the hand. This malignancy requires unique treatment considerations; the surgeon and patient must balance retention of maximal functional capacity of the hand and minimization of the risk of recurrence and metastasis. Digital-sparing and digital-sacrificing therapies should be considered. Chance for cure, recurrence and metastasis risk, cosmetic concerns, and functional concerns should be addressed on a case-by-case basis. We report a case of a fifty-three-year-old man with cutaneous squamous cell carcinoma of his non-dominant hand. Ulceration and rapid growth of a long-standing lesion of the dorsal hand prompted evaluation and treatment. Over the course of a year, three separate surgeries including digital amputations and metacarpal resections were required to manage this recurrent and invasive malignancy. Seven years post-operatively, our patient retained a full, painless range of motion arc of the left thumb and ability to grip utilizing a functional brace. Treatment of squamous cell carcinoma of the hand is not always straightforward. High rates of local recurrence require negative margins and diligent postoperative surveillance. Digital sparing therapy should be considered to minimize functional impairment and maximize cosmesis. However, aggressive treatment and amputation must be considered for advanced disease and if pursued, should focus on maximization of functional capacity as one of the treatment goals.

## INTRODUCTION

Squamous cells are thin, flat cells that comprise the outer layer of the skin, known as the epidermis. Genetic damage in these cell lines can lead to abnormal, uncontrolled cancerous growths (i.e., neoplasms, tumors).^1^ Squamous cell carcinoma (SCC) is the second most common skin cancer in the United States and most common malignancy of the hands, comprising approximately three quarters of all hand skin cancers.^2–4^

Chronic sun exposure predisposes the back of the hands to developing SCC.^5^ Historical factors such as occupations or lifestyles with significant sun exposure should also raise clinical suspicion when examining cutaneous lesions. Some examples of higher-risk occupations include lifeguards, landscapers, construction workers, and airplane pilots. Other known risk factors include fair skin complexion, advanced age, male sex, immunosuppression, prior exposure to human papillomavirus, chronic scarring conditions, familial cancer syndromes, and environmental exposures (e.g., agricultural chemicals, water contaminants).^3^

Squamous cell carcinomas of the hand generally prove more challenging and require special treatment considerations. Early identification and aggressive treatment are essential in preventing destruction and functional impairment. The anatomy of the hand is complex with numerous nerves, vessels, tendons, ligaments, and bones. For optimal management, SCC with invasion past the skin and into these deeper structures requires a multidisciplinary treatment team including dermatology, orthopaedic surgery, and oncology (if metastatic). Digit-sparing therapy is complicated by standard surgical margins of 4 mm. for low risk SCC and 6 mm. for high risk SCC.^5^ In the case of high-risk patients, various amputations may be the best options and should be considered early. In this paper, we present a unique case of recurrent infiltrating squamous cell carcinoma of the hand requiring multiple resections.

## CASE PRESENTATION

In 2010, a Caucasian male in his early 50’s presented to the orthopaedic hand clinic with the complaint of a flat, hyperpigmented lesion on the dorsum of his left third digit. This lesion had been present but asymptomatic for twenty-five years. After years of stability, the lesion began to ulcerate and bleed without any purulent drainage or local erythema. The patient did not immediately seek treatment, and the lesion grew at an alarming rate, doubling in size over four weeks.

The patient first presented to dermatology. Clinical exam revealed a verrucous (i.e., wart-like), ulcerated nodule approximately 30 mm. x 30 mm. with areas of ulceration measuring approximately 15 mm. x 15 mm. In office, biopsies of the left dorsal wrist and left third digit were taken. Biopsies demonstrated well-differentiated squamous cell carcinoma of both the third digit and wrist. Referral to an orthopaedic hand specialist was recommended for possible surgical evaluation due to the size of the lesions and concern for bony invasion.

This man’s past medical history was significant for pneumonia as well as severe refractory atopic dermatitis requiring multiple therapies including creams, ointments, moisturizers, injections, tar baths, restrictive diets, and skin grafting. His prior surgical history included a cervical epidural abscess requiring evacuation and fusion, septic right native knee, and tonsillectomy.

The patient’s social history was remarkable for a prior thirty-pack-year tobacco history and chemical exposure in his career as an automobile and aviation mechanic. He reported occasional social alcohol use and denied recreational drug use. He denied any family history of skin cancer, other malignancies, or notable dermatologic conditions.

The patient was referred to and evaluated by an orthopaedic hand specialist, several weeks later. The lesion was described to be nearly circumferential around the proximal interphalangeal joint of the third digit, with involvement of the third-fourth web space. Range of motion at this joint was severely restricted primarily in flexion, and sensation to light touch remained intact.

**Figure 1:**
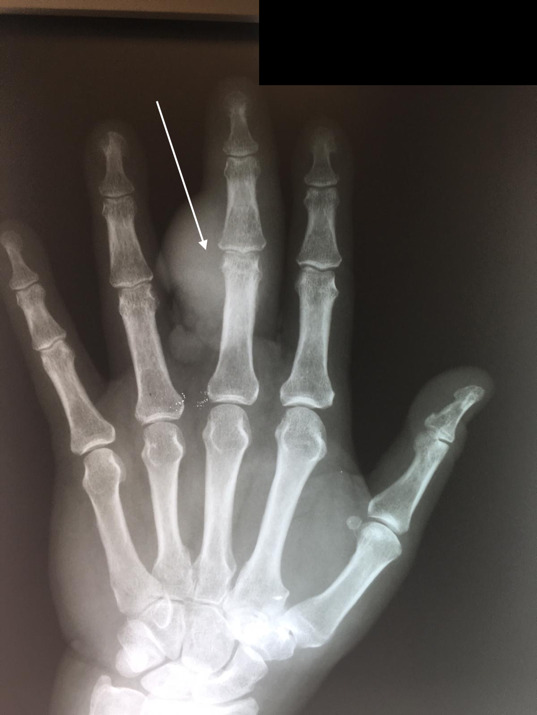
PA radiograph of left hand revealing the radiopaque lesion on the ulnar aspect of the third digit, centered at the PIP joint.

**Figure 2:**
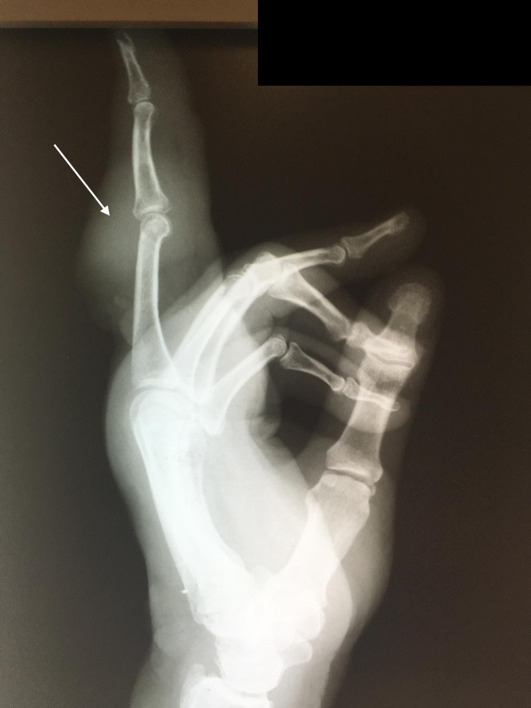
Lateral radiograph of the left hand revealing the radiopaque lesion on the dorsal aspect of the third digit, centered at the PIP joint.

In addition, evaluation revealed a raised dorsal hand mass near the wrist crease with ulceration. Two PA (Posterior-Anterior) and lateral radiographs (i.e., x-rays) of the left hand, taken at this time, demonstrated a radiopaque bone lesion on the ulnar aspect of the dorsal third digit, centered on the proximal interphalangeal (PIP) joint (Figures 1, 2). Magnetic resonance imaging (MRI) was obtained to assess for local osseous (i.e., bony) invasion. NOTE: The patient authorized the publication of all radiographic and photographic figures that will appear in this paper.

An MRI demonstrated a heterogeneously enhancing lobulated mass, located on the ulnar margin of the proximal middle finger phalanx and measuring 50 mm craniocaudal, 35 mm. Anterior-Posterior (AP), and 15 mm. in width. The mass closely apposed the extensor tendon complex of the third digit. There was no evidence of osseous invasion, bone marrow enhancement, or osseous enhancement. Surgical intervention was discussed with the patient.

The patient’s index (i.e., first) surgery was performed in August 2010. The surgery included amputation of left third digit with ray resection and excision of an approximately 30 mm. tumor from the dorsal hand. Specimens were collected and sent to pathology.

Pathologic description of the third digit was as follows: Pathology grossly described a mass overlying the middle and proximal phalangeal bones. This mass was 40 x 60 x 45 mm. There was a large ulceration on the mass measuring 60 x 30 mm. Microscopic examination revealed infiltrating, well-differentiated squamous cell carcinoma extending into the phalangeal bone. The proximal metacarpal margin was uninvolved. There was involvement of the skin, dermal, and subcutaneous tissues.

Pathologic description of the dorsal hand mass was as follows: Pathology grossly reported a large white 28 mm. lesion on the dorsal hand specimen. There was an ulceration measuring 15 mm. Microscopic examination revealed well-differentiated squamous cell carcinoma. There were malignant cells at the radial margin with questionable involvement of the ulnar margin. Tumor was present within 0.1 mm. of the deep margin of resection.

Positive surgical margins, or tumor identified pathologically at the border of the biopsy, accompanied by clinical indicators of recurrence prompted a second surgery. The second surgery was performed in November 2010. The surgery included left fourth digit and partial third and fourth metacarpal resection. A full thickness skin graft measuring approximately 20 mm x 60 mm was used with tissue harvested from the volar (or palmar) aspect of the ipsilateral (same) forearm.

Pathologic description of the second surgical specimen was as follows: Pathology grossly identified a subcutaneous grey-white mass adjacent to the proximal phalangeal bone, wrapping around the bone radially and extending near the dorsal surface. The mass was 25 x 25 x 30 mm. Microscopic examination revealed well to moderately-differentiated squamous cell carcinoma of depth 14 mm, of level V, involving the subcutaneum but not bone. Margins were at least 5 mm. The lesion was staged T2NxMx via the TNM Classification of Malignant Tumours.^6^ Proximal phalanx and metacarpal head were free of neoplasm.

**Figure 3:**
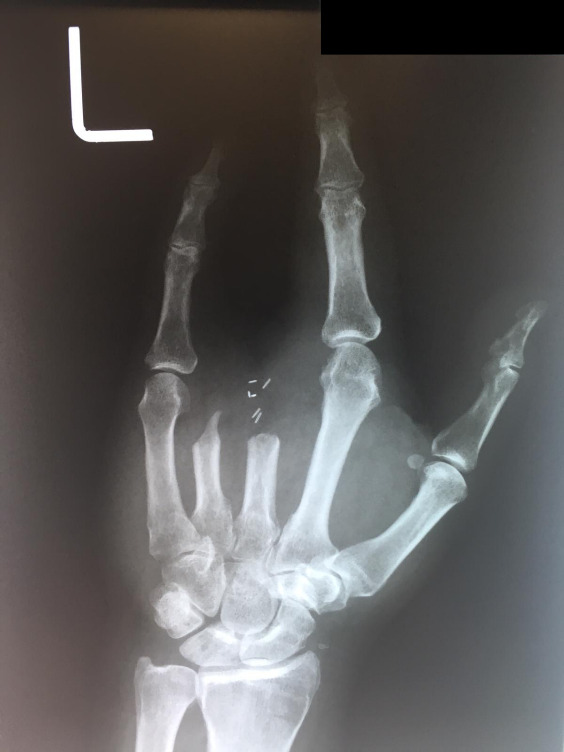
PA radiograph of the left hand after the second surgery but prior to the third surgery, demonstrating partial amputation of the third and fourth metacarpals.

The patient underwent follow up with dermatology during the postoperative period. Suspicion for recurrence prompted biopsies that confirmed SCC recurrence. A third surgery was performed in March 2011. The surgery included removal of second and fifth digit, partial metacarpal resection. A PA pre-surgical radiograph was taken at the clinic and included in this report (Figure 3). Pathology identified metastatic squamous cell carcinoma with negative surgical margins, or no tumor pathologically identified at edges of biopsy.

**Figure 4:**
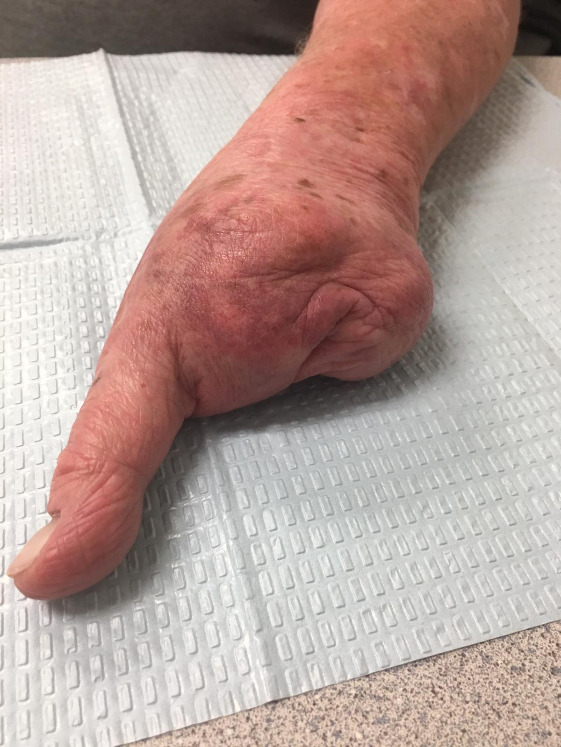
Clinical photograph of the left hand status-post metacarpal 2-5 partial resections. Well-healed surgical incision.

**Figure 5:**
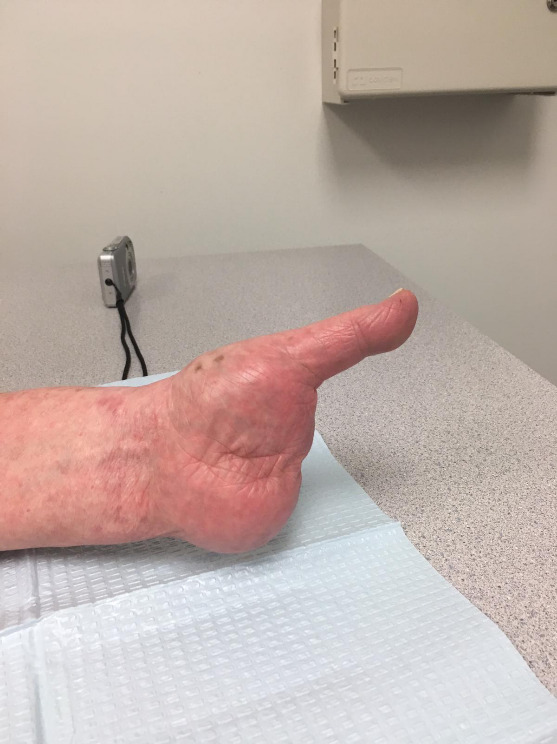
Clinical photograph of the left hand status-post metacarpal 2-5 partial resections. Thumb is completely preserved with a full, painless range of motion.

**Figure 6:**
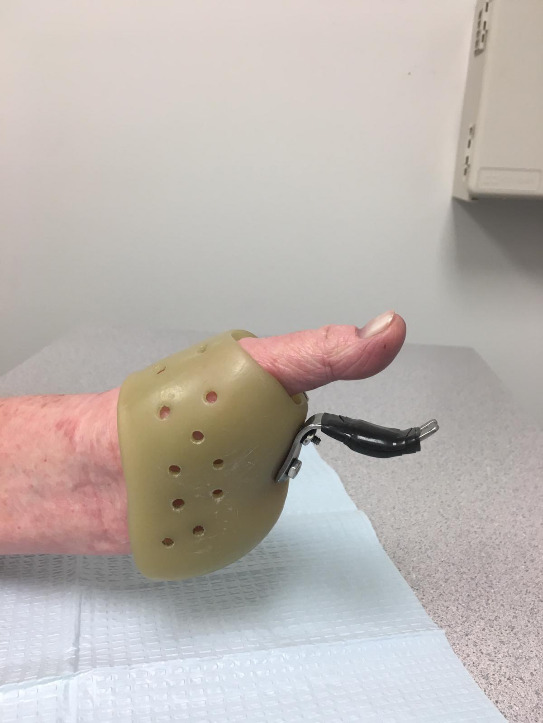
Clinical photograph demonstrating a well-fitted functional brace that allows for gripping between the thumb and brace.

As of the time of this publication (seven years after his most recent surgery), the patient denied phantom pain. He admitted to a full, painless range of motion and intact strength of his left thumb. The scar from his closure was well-healed (Figures 4, 5). The patient was unable to find a prosthetist able to fit him with a brace. With his background in engineering, he was able to assist a prosthetist with design and creation of a functional brace which assisted his grip (Figure 6). He has since been investigating myoelectric prosthetic hands and working with insurance companies to obtain one. The patient was retired when his SCC presented. He has continued to live at home alone and maintained his ability to complete activities of daily living.

## DISCUSSION

Squamous cell carcinomas on the skin of the hand are one of the more commonly encountered malignancies presenting unique clinical challenges. It is imperative that orthopaedic surgeons are familiar with this cutaneous malignancy due to the multidisciplinary approach required for optimal patient outcomes. A high index of suspicion should be utilized as presentation is variable and these lesions may appear inconspicuous and subsequently be misdiagnosed.^7^

There is still a paucity of similar cases documented in the literature. One 2009 case of verrucous carcinoma, a wart-like, well-differentiated SCC variant, on the palm of a male laborer in his early 30’s has been described.^8^ In this earlier case, extensive joint, muscle, and ligament neoplastic involvement prompted complete fourth ray resection with amputation of the whole finger and the entire associated metacarpal. This patient maintained functional use of the hand and continued his construction career.^8^

In suspected cases of cutaneous-osseous neoplasm, infection must be considered and vice versa as these disease processes share clinical and radiographic features but have considerably different treatment algorithms. Mirigliano, et al reported a lesion on the foot that was first mistakenly diagnosed as osteomyelitis.^7^ Clinical, laboratory, and MRI findings were unable to distinguish between infection and malignancy and ultimately pathology revealed the correct diagnosis of SCC. Next, a partial third ray resection was attempted but margins were inadequate with deep invasion of the tumor. Ultimately, the patient received a Lisfranc disarticulation, a procedure in which all five metatarsals and distal phalanges are sacrificed.^7^

In cases such as the patient discussed in this case report, early surgical intervention should be given adequate consideration, and the utility of digital-sparing versus digital-sacrificing therapy determined on a case-by-case basis. In another 2010 patient case treated conservatively for SCC of the hand, invasion into the bone had been masked by apparent lesion improvement. Osteolytic destruction extending to the proximal interphalangeal joint prompted ray resection.^9^

Some surgeons are moving towards preservative surgery in the treatment of SCC and other skin cancers of the hand.^10^ Digit-sparing surgical options include standard excision and Mohs micrographic surgery.^11^ However, the current literature still lacks a high quality of evidence comparing surgical interventions.^12^

Functional concerns and cosmesis are important factors to consider when attempting to optimize a patient’s chance for cure and quality of life. Providers’ desire to conserve function must be weighed against the risk of both metastasis and recurrence.^4^ As tumor invasion progresses from mid-dermis to bone, metastatic rates can approach 12.5%.^13^ In a 2012 retrospective review of 541 patients, Maciburko, et al reported a significantly increased rate of metastasis in SCC located on the web spaces and dorsal proximal phalanges.^4^ High rates of local recurrence also complicate cases of SCC.

In another 2013 cohort of 86 patients, Askari, et. al. reported a recurrence-free survival of 67% and 50% at 5 and 10 years, respectively.^14^ In this cohort, the average time until first recurrence was 4.1 years (range, 0.5-11 years), and identified risk factors included tumor location between the fingers, bilateral tumors, multiple tumors, and prior history of SCC.^14^

Diligent surveillance by both the patient and physician can help identify early signs and symptoms of cancerous recurrence and metastasis. There is a paucity of published data addressing the role of primary care physicians in diagnosing and screening for skin cancer. It is unknown how many patients first present to their primary care physician for a suspicious lesion. The current US Preventive Services Task Force does not recommend for or against routine skin cancer screening in patients without a known history of skin cancer.^15^ Thus, performing a full body skin examination is left to the discretion of the patient’s primary care physician. Low rates of skin cancer screening by primary care physicians was attributed to lack of training and lack of confidence in diagnosing skin cancer in a 2003 study.^15^ However, because most skin cancers are the result of cumulative UV radiation, primary care physicians are uniquely positioned to provide patients with preventative education addressing risk factors, most notably sun protection.^16^ Further studies are needed to explore the role of various providers across the healthcare system in diagnosing and treating cutaneous malignancies. Once a diagnosis of squamous cells carcinoma is made, it is recommended that patients undergo skin examinations with a board-certified dermatologist every 3-12 months for 2 years, then every 6-12 months for 3 years, then annually for life.^17^

## CONCLUSIONS

This case provides an example of the unique challenges and considerations physicians encounter when treating more severe SCC of the hand. The authors recommend considering both digit-sparing and digit-sacrificing therapy for these types of lesions and surgical decisions made on a case-by-case basis considering patient risk factors and goals of care.

The authors report no external funding source for this study.

The authors declare no conflict of interest.

Submitted for publication May 2020.

Accepted for publication August 2020.
